# On the Treatment of Scarlatina

**Published:** 1811-04

**Authors:** 


*To the Editors of the Medical and Physical Journal.*

GENTLEMEN,

JUDGING it to be the duty of medical men to acquaint each other with their most successful manner of treating diseases, particularly those which are frequently productive of great fatality; and as I am persuaded it not only tends to the improvement of medicine as a science, but will ultimately prove to be for the good of the community at large, I submit the following observations for publication in your highly esteemed Journal.

Observing the mortality which has lately occurred at Debenham, in Suffolk, occasioned by the Scarlatina Anginosa Maligna, I was led to recall to mind the plan from which I had experienced the most favourable results.

The treatment recommended and adopted in this often alarming malady is very opposite and curious; some practitioners enforcing a strict antiphlogistic plan, while others prescribe a tonic, invigorating, and even stimulating mode; though each of these may be useful, yet each may be carried to excess; hence both require limitations.

The practice from which I have experienced the greatest success, has been that of adopting the antiphlogistic plan at the commencement. Venesection as strongly, and no doubt deservedly, recommended by Mr. Hamilton, in my opinion, can only be had recourse to at the onset of the fever, when it may be considered only of an inflammatory kind, and in plethoric habits, which then may probably put a complete stop to its progress, or at least render it milder during its continuance. I speak cautiously of venesection, not having practised it sufficiently to give a decided opinion: but I can decide more positively concerning the use of emetics when given at the beginning of the attack. The remedies on which I chiefly rely, and may say, generally have administered with a very good effect, are frequent purges of a mercurial kind, and the saline mixture in the state of effervescence; I generally find it necessary to use some astringent gargle, and have usually prescribed the following—R. Cort. Cinchon, contus. ℥ss. Fol. rosæ rubr. ʒss. Coque in aq. font. ℔ss. per min. xv. cola et. adde. Acid. sulph. dil. gtt. lx. Sacch. alb. ℥ss. ft. garg. sæpe utend.

When nothing but debility remains I begin the tonic plan, and first give bark with the saline mixture, still in the state of effervescence.

I remain, Gentlemen,
Your obliged Servant,
T. F. R.

*London, Feb.* 19, 1811.

